# Descriptive epidemiology of the head and neck cancers in old patients

**DOI:** 10.3389/fonc.2023.1102236

**Published:** 2023-05-24

**Authors:** Gemma Gatta, Riccardo Capocaccia, Laura Botta

**Affiliations:** ^1^ Evaluative Epidemiology Unit, Department of Epidemiology and Data Science, Fondazione IRCCS Istituto Nazionale dei tumori, Milan, Italy; ^2^ Editorial Board, Epidemiol Prev, Milan, Italy

**Keywords:** head and neck cancer, elderly, epidemiology, population based cancer registries (PBCRs), incidence, survival, prevalence, world wide

## Abstract

**Background:**

In Europe, as in other high-income (HI) countries, quite half of the newly diagnosed patients with head and neck (H and N) cancers are older than 65 years of age and their proportion within the prevalent cases is even higher. Moreover, the incidence rate (IR) for all H and N cancers sites increased with age and the survival rate is lower in older patients (≥65), compared with younger patients (<65). The number of older patients affected by H and N cancers will increase because of the increase in life expectancy. The aim of the article is to provide an epidemiological description of H and N cancers in the elderly population.

**Material and methods:**

Incidence and prevalence data by time periods and continents were extracted from the Global Cancer Observatory. The survival information for Europe is obtained from the EUROCARE and RARECAREnet projects. In 2020, according to the results from these data, slightly more than 900,000 cases have been diagnosed with H and N cancers in the world, and approximately 40% were older than 65 years of age. This percentage was higher, reaching approximately 50% in the HI countries. The highest number of cases was in the Asiatic populations, while the highest crude IR was in Europe and Oceania. Among H and N cancers occurring in the elderly, laryngeal and oral cavity cancers were the most common, while nasal cavities and nasopharyngeal cancers were the rarest. This was true for all the countries, excluding some Asiatic populations, in which tumour of the nasopharynx was more common. The five-year survival rate in the European population was low in the elderly, compared with the younger for all H and N cancers, and it ranged from approximately 60% for both salivary-gland type and laryngeal to 22% for hypopharyngeal tumors. For the elderly, the conditional 5-year survival after surviving one year became more than 60% for many H and N epithelial tumors.

**Conclusion:**

The high variability in the H and N cancer incidence around the world is due to the distribution of the major risk factors which for the elderly are mainly alcohol and smoking. The reasons for low survival in the elderly are most likely due to the complexity of treatment, the late arrival of patients at diagnosis, and the difficult access to specialized centers.

## Introduction

The European population projections (1) suggest that the number and the percentage of people over age 65 will increase from 90.5 million in 2019 to 129.8 million in 2050. This population expansion will be accompanied by a marked increase in patients requiring care for disorders related to aging and will challenge both healthcare institutions and professionals.

Cancer is a disease of the elderly and head and neck (H and N) cancers, as the majority of epithelial solid cancers, occur in the elderly. It is a group of cancers that affects many different sites, from the oral cavity to the larynx, including the nasal cavities, the nasopharynx, and the hypopharynx. Alcohol, tobacco, several occupational risk factors, and viruses are the major factors responsible for the incidence of H and N cancers (2). According to the age of first exposure, the amount of exposure, and the latency time lag, the incidence of epithelial cancers increase with age. In the US, 70% of deaths from H and N cancer patients occur over the age of 70 (3). The prognosis of this group of cancers dramatically reduces with increasing age (4). Population aging, especially in Europe and western countries, will lead to an increasing prevalence of H and N cancer patients. Reducing exposure to the known risk factors is a feasible and almost mandatory public health task; however, curing the elderly affected by this cancer is problematic due to the comorbidity which affects these patients and their exclusion from clinical trial protocols.

The aim of this paper is to provide the major epidemiological indicators of the frequency (incidence and prevalence) and the outcome (relative survival) of H and N cancers with a focus on elderly patients, defined as those >65 (5, 6), in comparison with younger patients.

## Material and methods

We define H and N cancers according to the topography and morphology codes of the International Classification of Oncology (ICD-O) (7) used by the Global Cancer Observatory (GCO) (8) and the RARECAREnet project (9). The GCO defines H and N with the topography codes for lip and oral cavity to include the whole of the tongue (C00–06), salivary glands (C07–08), tonsils, oropharynx and nasopharynx (C09–11) and other ill-defined sites of the lip, oral cavity, pharynx (C12–14), and larynx (C32). The groups of epithelial H and N cancers according to the RARECAREnet project are nasal cavities and sinuses, nasopharynx, major salivary glands and salivary glands-type tumors, hypopharynx and larynx, oropharynx, oral cavity, and lip, including only the epithelial cancers as reported in reference no (9). We use GCO data for the incidence and prevalence estimations in 2020 (numbers and rates or proportions) and a specific section of the GCO, Cancer Incidence in Five continents, for incidence trends. We used the RARECAREnet search tool to provide data on the survival of H and N cancers in Europe.

Data sources were population-based cancer registries that were published periodically in the Cancer Incidence in Five Continents (CI5 Vol. X and CI5 plus), maintained by the International Agency for Research on Cancer. For the European survival data, the EUROCARE-5 project (10) was the principal source through the RARECAREnet (9) project that was focused on rare cancers and provided data on survival for the epithelial tumors of more detailed H and N cancer sites.

These data are available for public use.

Crude and age-standardized incidence rates were calculated and reported as 100 000 persons/year. Prevalence is reported as a 5-year prevalence; that is, people/patients alive within 5 years since their diagnosis, a measure including cured people, patients receiving primary treatment, people in clinical follow-up, and patients in recurrence or progression. To provide a measure of survival due only to cancer by removing the effect of mortality due to causes other than cancer the relative survival has been used.

A more detailed description of data and methods used for the calculation of the indicators are in (8) for incidence and prevalence and in (9) for survival.

## Results

### Incidence

**Figure 1:**
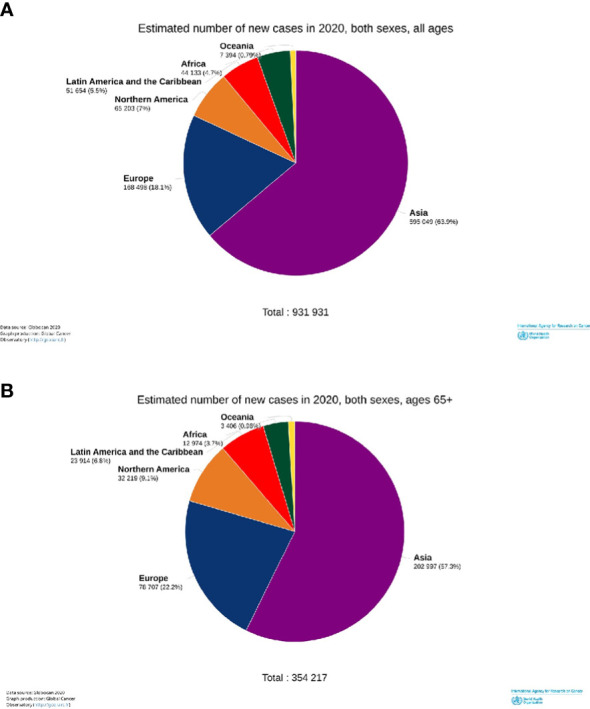
Estimated incident numbers of the head and neck cancers by continents, 2020. All cases **(A)** and elderly cases (65+ years old) **(B)**.

Worldwide, approximately 900 000 new H and N cancer cases were estimated to be diagnosed in 2020, of which 38% (354,217/931,931) occurred in patients older than 65 years of age (elderly) (**Figure 1**).

Restricting the analysis to the elderly (**Table 1**, right part), the burden, in terms of the raw number of new diagnoses, was the highest in Asia, while the risk (incidence rate) of being diagnosed with one of the H and N cancers was the highest in the populations of Oceania, Europe, and North America. Here, the age-standardized incidence rates ranged between 63 and 52 per 100 000/year (**Table 1**), while they were between 49 and 28 in other continents. An exception was nasopharyngeal cancer with the highest rates in Asia (6 per 100 000/year) and Africa (approximately 4) and rates of approximately 1 in other continents. Among H and N cancers, those of the lip and oral cavities showed the highest incidence rates between 10 (Africa) and 38 (Oceania). Laryngeal cancer had age-standardized rates between 8 (Africa) and 15 (Europe). Hypopharyngeal cancer showed a high risk in Asia and Europe (both approximately 6) and the lowest in Africa (approximately 1). Cancer of the oropharynx was high in North America, Europe, and Oceania, and approximately the double than in Asia and Africa. Cancer of the salivary glands was one and a half times higher in continents with high incidence (North America and Oceania) than in other continents. In the elderly, H and N incidence rates were between two/three times higher in all the continents, compared to cases of adults between 35 and 64 years old (**Table 1**). This holds for each single cancer site, with the exception of the nasopharynx with an incidence ratio of 1:3 between the elderly and adults. Furthermore, the incidence ratios between the elderly and younger patients were highest in the LAC, except for the oropharynx (highest in Asia) and hypopharynx for which the difference was highest in Oceania.

**Table 1:** Estimated number of new cases and incidence rates for head and neck cancers by site and continent in two age groups, 35 to 64 years old and 65+ years old, in 2020.

	Continent	35 to 64 years old			65+ years old			
		*Number*	Crude Rate	ASR (World)	*Number*	Crude Rate	ASR (World)	ASR ratio between the two age groups
Lip and oral cavity	Oceania	*2 260*	15.2	14.6	*2 063*	37.9	37.7	2.6
	Europe	*32 006*	10.3	9.3	*32 772*	22.9	22.8	2.5
	Northern America	*12 699*	9.1	8	*14 293*	23.1	22.9	2.9
	Asia	*148 737*	8.8	8.9	*81 112*	19.7	19.5	2.2
	LAC	*9 072*	4	4.1	*8 134*	13.9	13	3.2
	Africa	*8 161*	2.7	3.2	*4 569*	9.7	9.8	3.1
	Total	212935	8	8	142 943	19.6	19.3	2.4
salivary glands	Northern America	*1 975*	1.4	1.3	*3 145*	5.1	4.6	3.5
	Oceania	*206*	1.4	1.4	*244*	4.5	4.1	2.9
	Europe	*3 700*	1.2	1.1	*5 770*	4	3.6	3.3
	Africa	*2 529*	0.85	0.94	*1 414*	3	3	3.2
	LAC	*1 913*	0.84	0.86	*1 929*	3.3	3	3.5
	Asia	*14 809*	0.88	0.88	*10 273*	2.5	2.5	2.8
	Total	25 132	0.94	0.94	22 775	3.1	3	3.2
oropharynx	Northern America	*8 544*	6.1	5.4	*5 433*	8.8	9.6	1.8
	Europe	*18 052*	5.8	5.2	*11 103*	7.8	8.8	1.7
	Oceania	*733*	4.9	4.7	*434*	8	8.8	1.9
	LAC	*5 286*	2.3	2.4	*3 364*	5.7	5.5	2.3
	Asia	*25 435*	1.5	1.5	*15 848*	3.9	3.8	2.5
	Africa	*1 982*	0.66	0.77	*725*	1.5	1.5	1.9
	Total	60 032	2.2	2.2	36 907	5.1	5.2	2.4
nasopharynx	Asia	*77 591*	4.6	4.6	*24 965*	6.1	6.1	1.3
	Africa	*5 859*	2	2.1	*1 646*	3.5	3.6	1.7
	Europe	*3 061*	0.98	0.93	*1 783*	1.2	1.3	1.4
	LAC	*1 046*	0.46	0.47	*773*	1.3	1.3	2.8
	Northern America	*1 267*	0.9	0.85	*711*	1.1	1.2	1.4
	Oceania	*157*	1.1	1	*56*	1	1.1	1.1
	Total	88 981	3.3	3.3	29 934	4.1	4.2	1.3
hypopharynx	Europe	*11 185*	3.6	3.2	*7 785*	5.4	6.1	1.9
	Asia	*32 100*	1.9	1.9	*24 547*	6	5.9	3.1
	Oceania	*102*	0.69	0.65	*141*	2.6	2.6	4.0
	Northern America	*1 155*	0.82	0.71	*1 301*	2.1	2.2	3.1
	LAC	*1 283*	0.56	0.58	*1 123*	1.9	1.9	3.3
	Africa	*1 266*	0.42	0.48	*650*	1.4	1.4	2.9
	Total	47 091	1.8	1.8	35 547	4.9	4.9	2.7
larynx	Europe	*20 291*	6.5	5.7	*19 494*	13.6	14.6	2.6
	LAC	*7 417*	3.3	3.3	*8 591*	14.6	14.4	4.4
	Northern America	*6 154*	4.4	3.8	*7 336*	11.9	12	3.2
	Asia	*56 879*	3.4	3.4	*46 252*	11.2	11.2	3.3
	Oceania	357	2.4	2.3	*468*	8.6	8.5	3.7
	Africa	*5 523*	1.9	2.2	*3 970*	8.4	8.4	3.8
	Total	96 621	3.6	3.6	86 111	11.8	11.9	3.3
H and N cancers	Oceania	3 815	25.6	24.7	3 406	62.5	62.7	2.5
	Europe	88 295	28.4	25.4	78 707	55	57.3	2.3
	Northern America	31 794	22.7	20.0	32 219	52	52.4	2.6
	Asia	355 551	21.1	21.2	202 997	49.3	49.1	2.3
	LAC	26 017	11.5	11.7	23 914	40.8	39.1	3.3
	Africa	25 320	8.5	9.7	12 974	27.5	27.8	2.9
	Total	530 792	19.8	19.8	354 217	48.6	48.5	2.4

Crude, standardized (ASR) incidence rate per 100 000/year, and the ratio between standardized incidence rates for 35 to 64 and 65+ years old. Continents are ranked by 65+ years old crude rate.

LAC, Latin America and the Caribbean.

**Figure 2:**
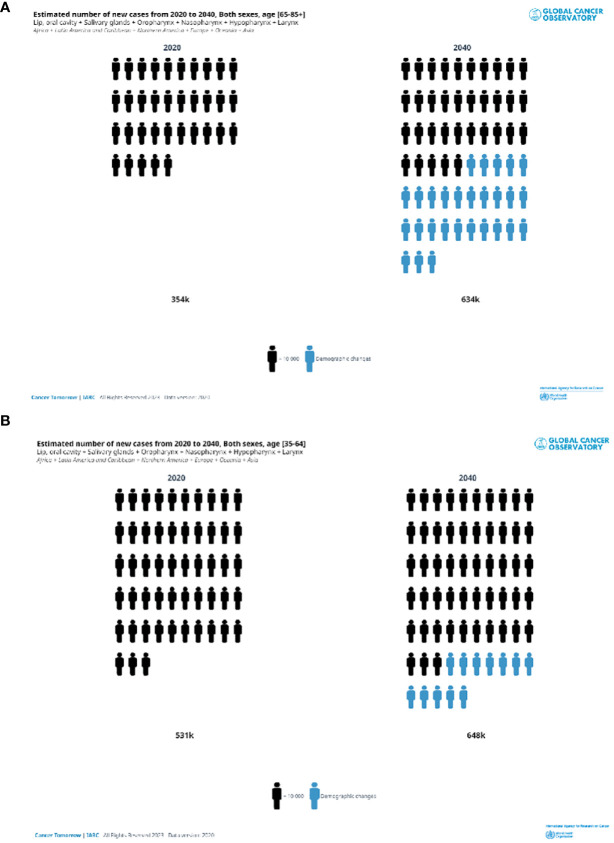
Estimated number of head and neck new cases in 2020 and 2040. Both sexes, in two ae groups **(A)** 65+years old, **(B)** 35–64 years old.

**Figure 3:**
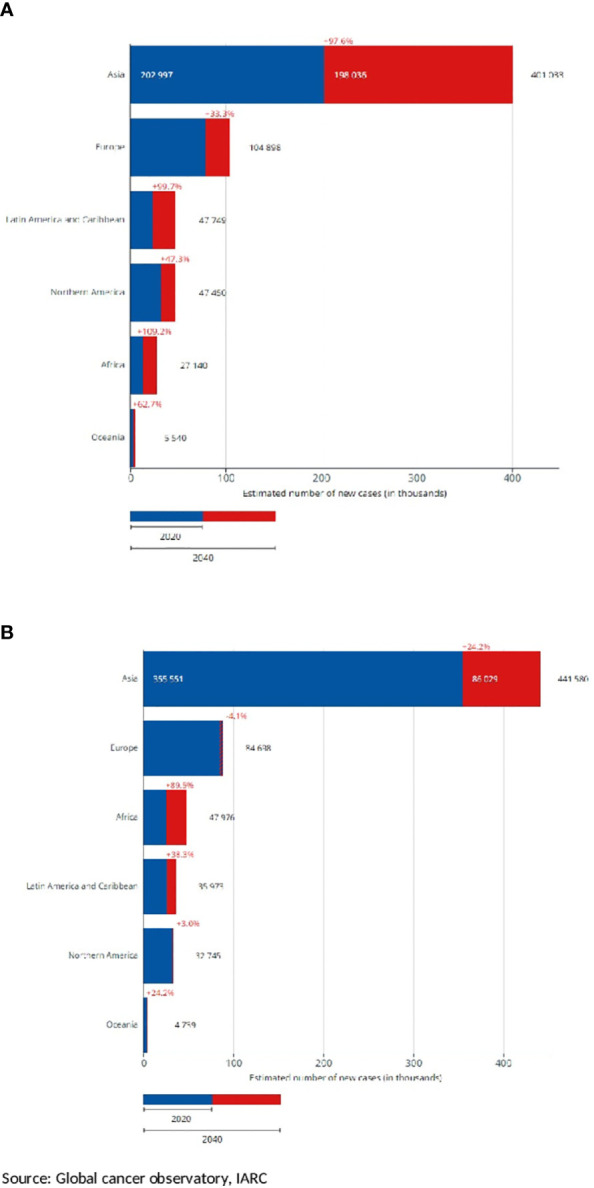
Estimated number of new head and neck cancer cases by continent, projection to 2040, and diffences with 2020. Both sexes, in two age groups **(A)** 65+ years old, **(B)** 35–64 years old.

**Figure 2** shows that according to the estimates of the GCO, for the elderly, the number of H and N cancer yearly new diagnoses will double from that of 2020 by 2040. Increases will be observed in all the continents (**Figure 3**). The highest increase in elderly cases (>95%) will be in Africa, Asia, and the LAC (**Figure 3A**) and the lowest will be in Europe (33%). Overall, the increase was higher for nasopharyngeal cancer (87%) and lower for the oropharynx (67%), results not shown. The burden in 20 years for younger patients (35 to 64 years of age, **Figure 3B**) will be lower compared to elderly patients. Indeed, it will be slightly less than 25% in almost all the continents (**Figure 3B**) except LAC (38%) and Africa (89%). The increase by cancer site was similar across cancer sites: from 17% for oropharyngeal cancer to 25% for nasopharynx, results not shown.

**Figure 4:**
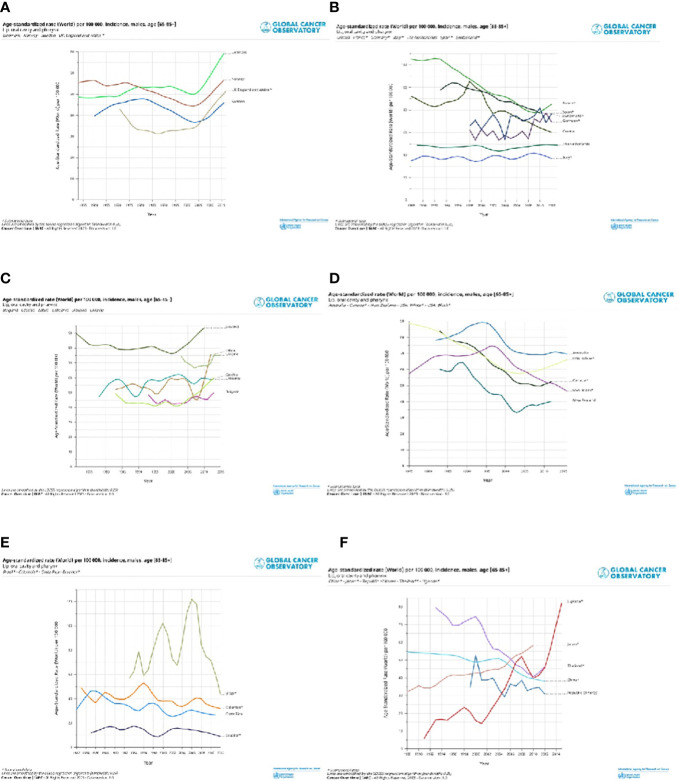
Age standardised (World) per 100,000, incidence trend, age 65+, by different countries in North Europe and UK **(A)**, Central and South Europe **(B)**, East Europe **(C)**, non-European HI countries **(D)**, Latin America and Caribbean countries **(E)** and Asiatic countries and Uganda **(F)**. Lip, oral cavity and pharynx, males.

**Figure 5:**
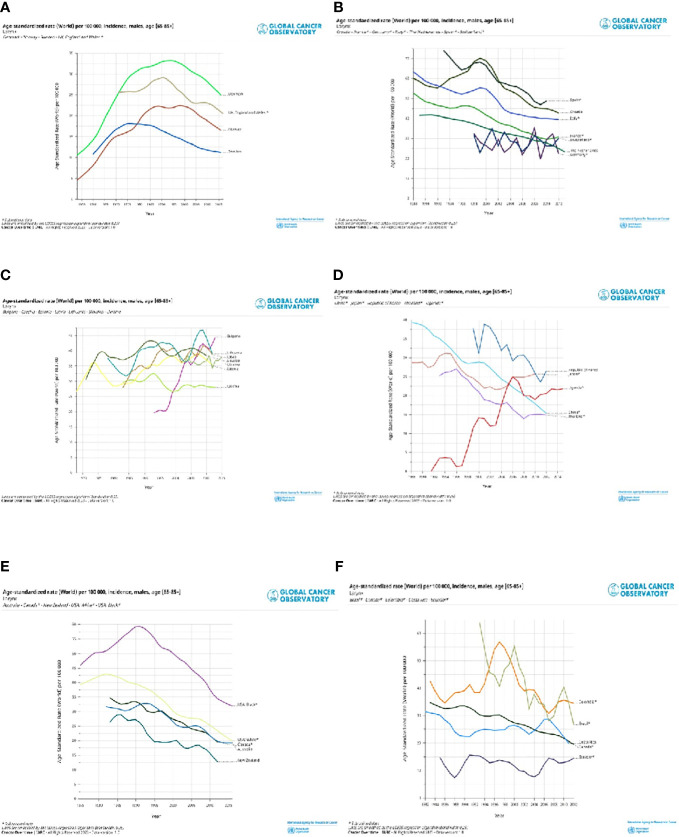
Age standardised (World) per 100,000, incidence trend, age 65+ , by different countries in North Europe and UK **(A)**, Central and South Europe **(B)**, East Europe **(C)**, non-European HI countries **(D)**, Latin America and Caribbean countries **(E)** and Asiatic countries and Uganda **(F)**. Larynx, males.

Selected registries with a long period of registration and incidence trends by countries are shown for cancers of the lip, oral cavity, and pharynx (**Figure 4**) and the larynx (**Figure 5**). Countries are grouped into North, Central South, and East of Europe (a-c), non-European HI countries (d), LAC countries (e), and Asiatic countries, and Uganda (f). The trends are reported only for old men (65+), in which the incidence is very high. For the lip, oral cavity, and pharynx incidence rates, (**Figure 4**) after a quite stable or reducing trend, there were increases in some populations, except the LAC, the US Black, Australia, Israel, China, and some central and southern European countries. By contrast, incidence trends were decreasing for the larynx (**Figure 5**) with different speeds by country. Countries with a long period of registration showed a peak across90s, in the US, New Zeeland, and some European countries. Incidence was increasing over time in Uganda, Bulgaria, Lithuania, and India.

### Survival

From the RARECAREnet project, **Table 2** shows the 5-year relative survival (RS) of H and N cancers in elderly and adult patients (25 to 64 years of age). For all the entities, 5-year RS was better in younger patients, and differences between the adults and elderly patients were important for the epithelial tumor of the nasopharynx, major salivary glands, oropharynx, and the salivary glands-type tumors (>10 points absolute difference). However, after surviving for one year, that is excluding cases died within one year since diagnosis and most likely presenting at an advanced stage, the 5-year RS for the elderly became more than 60% for many H and N epithelial tumors. The differences between the two age groups are reduced, comparing conditional survival for all H and N cancers and even achieving equality between the two age groups for laryngeal and oral cavity epithelial tumors (**Table 2**). The progress in the 5-year relative survival is available from the RARECAREnet website for all patients, without distinguishing by age. For almost all H and N cancer entities, improvement was significant except for the larynx. The last column of **Table 2** shows that the best improvements occurred for the epithelial tumor of the oropharynx (+7%) and the epithelial tumors of the nasal cavity and sinuses (+5%).

**Table 2:** Number of cases (No.) and 5-year Relative Survival (RS%) with 95% CI for epithelial H and N cancers in Europe (RARECAREnet) and years of diagnosis 2000 to 2007.

H and N cancers	Age group						
	25 to 64			65+			
	No.	5-year RS (95% CI)	CS(%)	No.	5-year RS (95% CI)	CS(%)	5-year survival progress*
Epithelial tumors of the nasal cavity and sinuses	3,055	53 (51-55)	67	3,777	42 (40-45)	61	+5%
Epithelial tumors of the nasopharynx	4,791	55 (53-56)	67	2,157	31 (29-34)	51	+2%
Epithelial tumors of major salivary glands	6,703	70 (69-71)	80	7,625	50 (49-52)	67	+2%
Salivary gland-type tumors of the head and neck	3,574	71 (70-73)	80	2,992	60 (58-63)	73	+2%
Squamous cell carcinoma of the hypopharynx	13,136	27 (26-27)	42	6,739	22 (21-24)	39	+4%
Squamous cell carcinoma of the larynx	38,749	62 (61-62)	72	33,155	59 (58-60)	71	0%
Epithelial tumors of the oropharynx	35,409	44 (43-44)	59	15,411	34 (33-35)	54	+7%
Squamous cell carcinoma of the oral cavity	31,440	50 (49-50)	64	22,688	46 (45-47)	65	+4%

CS= is the conditional 5-year survival, the probability of surviving an additional 4 years on the condition that the patient has survived 1 year.

*=from 1999 to 2002 and 2005 to 2007, all ages.

### Prevalence

An estimation of cancer prevalence by age, defined as the number of people living at a given time point with a diagnosis of cancer, is available from GCO, for 2020. Cancer prevalence mainly depends on incidence and survival. **Table 3** shows the 5-year prevalence in terms of the number of cases and proportion per 100,000. In 2020, the number of prevalent cases is the highest in Asia, especially for <65 years old people due to the large population covered. By contrast, the proportion was high (>160 per 100,000) in Europe, Oceania, North America, and Asia. The prevalence in the elderly was more than two times higher than in the young people. The ratio between the elderly and adult prevalence was high, accounting for more than three for Africa and LAC.

**Table 3:** Estimated number of 5-years prevalent cases in 2020 (number and proportion), salivary glands, oropharynx, nasopharynx, hypopharynx, larynx, lip, oral cavity, both sexes, by age group and continent.

Age	35 to 64 years old		65+ years old	
Continents	Number	Proportions	Number	Proportions
Europe	251 878	81	241 000	168,4
Oceania	10 864	73	10 445	191,7
Northern America	100 773	72	105 843	171
Asia	844 317	50,2	510 634	124,1
Latin America and the Caribbean	67 704	29,8	65 312	111,4
Africa	52 308	17,5	28 416	60,3

*** Proportions per 100 000

## Discussion

The paper provides the incidence and prevalence estimates of H and N cancers in 2020 across the continents and patients’ survival in Europe. The burden and the outcome by different H and N cancer sites are also separately given for two large age groups of patients (<65 and 65+ years) in order to focus on the impact of the disease on the elderly. With some differences by continent, the burden for the old patients was high everywhere. In Europe, the outcome (5-year relative survival) dramatically decreased by age at diagnosis, mainly for the epithelial tumors of the nasal cavities, nasopharynx, salivary glands, and oropharynx. For these cancer sites, the absolute difference between 25 to 64 and 65+ years of age patients varied between 10 and 23 percent (see **Table 2**).

The geographical variation of the H and N cancers incidence rates depends on the distribution in populations of the major risk factors of these malignancies, the most important being tobacco and alcohol with tobacco smoking and alcohol consumption having synergistic effects (2). Among the other factors, which are mainly site-specific, there are radiations, occupational exposures, and viruses. The prevalence of several risk factors, like tobacco, asbestos, and other carcinogenic agents mainly involved in occupational activities (11), were higher for the older cohorts of patients which in turn had a longer time of exposure than the younger cohorts. The global prevalence of daily smoking consistently fell since 1990, reflecting concerted efforts to implement strong tobacco control policies and programs (12). The reductions were especially pronounced in high Socio-Demographic Index countries and Latin America. Alcohol consumption is currently and historically the highest in Australia, some European regions (especially in central and eastern Europe), and Russia (13)

Diet is crucial for all H and N cancers (2, 14). The updated WCRF confirms the high risk due to alcohol and adult obesity, and there was a reduction of risk in the greater intake of non-starchy vegetables (14). For nasopharyngeal cancer, there is strong evidence of risk from consuming Cantonese-style salted fish and some evidence from consuming red meat and processed meat. Cantonese-style salted fish contains nitrosamines and nitrosamine precursors which have been shown to induce the development of cancer. One can assume that a diet including Cantonese-style salted fish was more common in the old cohort of cases since this type of diet has reduced (14).

The human papillomavirus (HPV) infection plays an increasing role in oropharyngeal cancer incidence.

In 2018, high-risk HPVs were found in almost half of the oropharyngeal cancers in more developed countries, being higher in North America and Northern Europe than in Southern Europe (15). In the few less developed countries with available data, high-risk HPVs were found in no more than 30% of cases. Furthermore, it is likely that the proportion of attributable fraction of HPV in oropharyngeal cancers is larger among non-smokers than among current smokers (16).

The number of new cases attributable to HPV infection was estimated to be 1/3 among the patients diagnosed at ≥70 years compared to the younger cohorts of cases (17).

Epstein-Barr virus (EPB) infection is a major player in nasopharyngeal cancers. The diffusion pattern of the virus around the world is consistent with the different incidences of the disease: higher in South-eastern Asia (15) compared to other continents. Actually, the fraction of NPC attributable to EBV in these areas is nearly 100% (15).

IARC has classified several occupational exposures as carcinogens for H and N cancers. The main factors were asbestos, wood and leather dust, metals/metalloids, formaldehyde, and radiation substitute with the right citation (11). The evolution of industrial practices and changes in legislation explain the higher exposition in the elderly.

The outcome of this study reported 5-year relative survival figures in the European populations and showed poor figures for the elderly. Comorbidity and incidence of second cancers related to tobacco and alcohol increase with age and are generally associated with worse survival. Standard treatment protocols are less available for elderly patients, who are usually not eligible for clinical trials because of age. Elderly patients present at a more advanced stage at diagnosis (4) and this may lead to difficulty in treating cases. The distribution of sub-site or histotype across populations due to different exposure to the major risk factors is also related to the aggressiveness of the disease. Alcohol and tobacco are related to different subsites of H and N cancer with different risks of dying (18). Furthermore, oropharyngeal cancer HPV positive presents a better survival than the HPV negative ones, suggesting a difference in the natural history of the disease between elderly and younger patients the latter more affected by HPV positive cancer.

The management of H and N cancers is complex and multimodal, and it requires a multidisciplinary approach. Access to appropriate treatment is therefore not easy. The considerably higher conditional survival of the elderly between 1 and 5 years with respect to the 5-year relative survival from diagnosis indicates a remarkable risk of death in the first year, presumably related to cancers diagnosed at an advanced stage. It is the case of laryngeal localization in which more than 50% of cases are diagnosed at an early stage, while they are less than 20% in hypo and oropharynx cancers (4). Curative treatment will be more effective and completely achievable in localized diseases. The epithelial tumors of major salivary glands and salivary-gland type tumors, also described in the paper by Colombo et al. of this special issue (19), stated that good prognosis histotypes and early-stage diagnosis is less common in the elderly with respect to the younger patients. Furthermore, unspecified histotypes presented more frequently in the elderly. Proper pathological and molecular analyses should be carried out for diagnostic and therapeutic purposes and unspecified histotypes could therefore be an indicator of suboptimal pathological diagnosis which is the prerequisite for appropriate treatment planning. Nasopharyngeal cancers showed a very low outcome in elderly patients (see **Table 2**), in which presentation at stages III and IV was described in more than 80% of cases by a SEER study (20). The first line of treatment for locoregionally advanced nasopharyngeal cancers should be concurrent chemo-radiotherapy (CCRT) and the study showed that elderly patients can benefit also when presented in advanced stage (T3 or N2 or stage III). Nasal cavities cancer is one of the most fatal among the H and N cancers. Treatment strategies consist of either radiotherapy, surgery, or a combination of the two; however, several studies suggest that T1 tumors can be successfully handled by a single treatment modality. The stage at diagnosis is probably still the most important parameter in terms of survival from nasal cavities cancer and the establishment of broad multidisciplinary teams is thus crucial for proper treatment planning, mainly in elderly patients (21).

The rising oropharyngeal cancer incidence and outcome in elderly patients in the US (22) was attributed to the rapidly increasing prevalence of HPV in elderly patients. Elderly patients with HPV-positive oropharyngeal squamous cell carcinoma (OPSCCs) may tolerate multimodality therapy better than the lifelong tobacco and alcohol users who accounted for the majority of patients in historical head and neck cancer trials. Given the excellent locoregional outcomes observed for HPV-positive OPSCCs, deintensification strategies may help in decreasing toxicity and maintaining acceptable oncologic outcomes.

H and N tumors are rare, and studies have demonstrated the value of them being treated in large-volume specialized centers (23). We believe that the benefits of high-volume centers must be made accessible to all patients with H and N cancers for optimal outcomes to be achieved, especially for those diagnosed in elderly patients with possible comorbidities that affect the application of standard and complete treatment protocols. Networks of hospitals are an alternative, with small or local centers, close to patients’ homes, linked to high-volume centers in a network arrangement to avoid moving discomfort, mainly for elderly patients. For this reason, each country should identify the institutions that can provide the best treatment. In Europe, this task had been done in agreement with the European Reference Networks which are virtual networks involving healthcare providers across Europe, with the aim to tackle complex or rare diseases and conditions that require highly specialized treatment and a concentration of knowledge and resources (24).

## Author contributions

GG drafted the article. GG, RC, LB performed the online search for the results. All authors contributed to the article and approved the submitted version.
